# The Relationship Between Self-Control and Internet Addiction Among Students: A Meta-Analysis

**DOI:** 10.3389/fpsyg.2021.735755

**Published:** 2021-11-24

**Authors:** Shiqi Li, Ping Ren, Ming Ming Chiu, Chenxin Wang, Hao Lei

**Affiliations:** ^1^Graduate School of Education, Peking University, Beijing, China; ^2^School of Education, Guangzhou University, Guangzhou, China; ^3^Department of Special Education and Counselling, The Education University of Hong Kong, Tai Po, Hong Kong SAR, China; ^4^Institute of Curriculum and Instruction, Faculty of Education, East China Normal University, Shanghai, China

**Keywords:** self-control, meta-analysis, internet addiction, moderator analysis, students

## Abstract

As past studies of self-control and Internet addiction showed mixed results, this meta-analysis of 83 primary studies with 80,681 participants determined whether (a) these students with less self-control had greater Internet addiction, and (b) age, culture, gender, Internet addiction measures, or year moderated these relations. We used a random-effects meta-analysis of Pearson product-moment coefficients *r* with Fisher’s *z*-transformation and tested for moderation with the homogeneity tests. The results showed a positive link between impulsivity and Internet addiction (*r* = 0.371, 95% CI = [0.311, 0.427]) and a negative link between restraint and Internet addiction (*r* = −0.362, 95% CI = [−0.414, −0.307]). The moderation analysis indicated that the correlation between impulsivity indicators and greater Internet addiction was stronger among undergraduates (18–22 years old) than among adolescents (10–17 years old). Furthermore, the negative link between a restraint indicator and Internet addiction was greater (a) among students in East Asia than those in Western Europe/North America, (b) among males than females and (c) when using the Internet addiction measures GPIUS or IAT rather than CIAS. Hence, these results indicate a negative link between self-control and Internet addiction, and this link is moderated by age, culture, gender, and Internet addiction measure.

## Introduction

The Internet has become an indispensable part in people’s daily life, which brought us both positive and negative effects. One of the typical negative phenomena concerning excessive use and abuse of computers or the Internet is Internet addiction. A meta-analysis has found that an estimated 6% of adolescents worldwide have suffered from Internet addiction (Cheng and Li, 2014). It is an impulse-control disorder that does not involve an intoxicant (Young, 1998a, 2004), which has been characterized by poorly-controlled preoccupations, urges, or behaviors regarding Internet access that lead to impairment or distress (Shaw and Black, 2008). Some studies have indicated that Internet addiction disrupts adolescents’ time management, especially daily sleep and exercise routines, which weakens their immune system (Rosen et al., 2014), interferes with their social relationships (Tsitsika et al., 2014), hinders academic achievement (Chou et al., 2005), and increases their likelihood of depression symptoms (Lam and Peng, 2010).

*Self-control* is defined as an inhibition capacity to regulate one’s thoughts, emotions, and behaviors in the face of external demands (DeLisi, 2014). Given the dual-systems model of self-control (Hofmann et al., 2009) and some empirical evidence like the Brief Self-Control Scale (Dvorak and Simons, 2009; Maloney et al., 2012), the domain of self-control can be divided into two dimensions: restraint and impulsivity (Carver, 2005). When facing temptation, individuals need to deal with two opposite forces, namely, the reflective force that promotes individuals to behave reasonably and the impulsive force that encourages individuals to satisfy their desires (Hofmann et al., 2009). Thus, restraint and impulsivity are distinct processes affecting self-regulatory outcomes (Strack and Deutsch, 2004; Carver, 2005; Hofmann et al., 2009). According to impulsiveness theory (Ainslie, 1975), as students with greater control over their emotions, thoughts, and behaviors are often less impulsive (Peluso et al., 1999), they might engage in fewer short-term behaviors, such impulsive Internet addiction, that conflict with their long-term goals. However, other researchers argue that self-control is unrelated to Internet addiction (Iftikhar and Tariq, 2014; Öğütçü et al., 2016), and past studies of this possible link showed mixed results (Kim et al., 2008; Tao and Li, 2009; Błachnio and Przepiorka, 2016), possibly due to moderator effects. To synthesize these results, this meta-analysis of past studies during 2002–2019 determines (a) the overall link between self-control and Internet addiction and (b) whether culture, age, gender, Internet addiction measure or year moderates this link.

## Self-Control and Internet Addiction

We first discuss why self-control might be linked to Internet addiction. Then, we consider how culture, age, gender, Internet addiction measures or publication year might moderate such links.

### Restraint, Impulsivity, and Internet Addiction

According to impulsiveness theory (Ainslie, 1975), some students have more restraint than others and are less impulsive (Peluso et al., 1999), so they are more likely to sacrifice short-term amusement to invest effort toward attaining long-term goals. For example, students with less restraint than others are more likely to avoid studying for the next day’s test by watching videos on the Internet. (Not all uses of the internet are harmful; for example, a student can search for relevant information on the Internet for an essay comparing theocracy in Iran and monarchy in North Korea). By contrast, students with less restraint than others are more likely to make decisions based primarily on short-term benefits (such as watching an Internet video) rather than on investments for long-term benefits, like studying for a test (Metcalfe and Mischel, 1999; Duckworth and Steinberg, 2015). If such students with less restraint have few other means to satisfy their emotional needs, they might increasingly use the Internet to do so (Ponton and Rhea, 2006). Thus, their repeated decisions to enjoy Internet use can foster Internet addiction (Baumeister et al., 2010; Shek and Lu, 2012). In contrast, other researchers argue that such decisions need not be impulsive, so that self-control might be unrelated to Internet addiction (e.g., Hur, 2006). In other words, when students are addicted to the Internet, they make rational decisions because they know the characteristics of the Internet as anonymity, convenience, and escape, not because of impulse (Young, 1998a).

Past studies of self-control and Internet addiction have shown mixed results. Some studies have revealed that restraint indicators (e.g., impulse control and resist temptation) are negatively related to Internet addiction (Larose et al., 2003; Kim et al., 2008; Li et al., 2014). Also, other studies have demonstrated that impulsivity indicators (e.g., impulsivity and temper) are positively related to Internet addiction (Gao and Zhao, 2009; Tao and Li, 2009).

However, many other studies have shown no significant relationship. Some studies have found no significant link between restraint indicators (e.g., self-control, self-regulation, etc.) and Internet addiction (Iftikhar and Tariq, 2014; Lee and Cho, 2015; Błachnio and Przepiorka, 2016). Likewise, other studies have shown no significant link between impulsivity indicators (e.g., impulsivity, dyscontrol, etc.) and Internet addiction (Choi et al., 2013; Öğütçü et al., 2016; Zhou, 2017). Although moderators (e.g., culture, age, and gender) might account for some of these differences in results, most studies did not test for them (Teng et al., 2014). Also, some of these differences in results might stem from using different measures (restraint vs. impulsivity indicators, different Internet addiction measures) or across the years of the studies. Hence, we consider possible moderation effects in the next section.

### Moderation: Culture, Age, Gender, Internet Addiction Measures, and Year

As many studies include information on the participants’ culture, age, gender, internet addiction measures and year, our meta-analysis can test for their moderation effects. Hence, we discuss the potential moderation of each variable.

#### Culture

A society’s cultural values might moderate the link between self-control and Internet addiction. While some societies highlight short-term goals [e.g., United States (US)], others encourage attention to long-term goals (e.g., China; Hofstede et al., 2008). As emphasis of long-term goals can reinforce their importance to students with restraint, they might attend to and devote effort to these long-term goals (Dewitte and Cremer, 2001), thereby further reducing the attractiveness of short-term Internet amusements and the likelihood of Internet addiction. For example, the correlation between restraint and Internet addiction is weak among university students in the United Kingdom (Mehroof and Griffiths, 2010) but much stronger among those in mainland China (Du, 2013). However, other studies show strong negative links in the United States (Khang et al., 2013) and the Netherlands (Van Deursen et al., 2015) but weak negative links in South Korea (Lee and Cho, 2015).

In contrast, students with low self-control are likely to ignore such long-term goals, so this cultural value is not likely to influence them (Metcalfe and Mischel, 1999). Hence, the link between impulsivity indicators and Internet addiction is likely substantial across countries. For example, studies show moderate to large links between impulsivity indicators and Internet addiction in Australia (Chou and Ting, 2003), Canada (Davis et al., 2002), mainland China (Bai and Xu, 2009), Hungary (Demetrovics et al., 2016), and South Korea (Choi et al., 2014).

Thus, a society with a long-term orientation might further strengthen the negative link between restraint and Internet addiction. However, this cultural value might not moderate the positive link between impulsivity and Internet addiction. Hence, this meta-analysis tested whether the link between self-control and Internet addiction differed across countries.

#### Age

Among low self-control students, younger students’ impulsivity might render them more vulnerable to Internet addiction (Wang et al., 2017), but university students’ loss of friendships and parental monitoring might increase their loneliness and likelihood of seeking solace on the Internet (Throuvala et al., 2019; Teng et al., 2020). On the one hand, as children grow into adults, their brains develop; specifically, their *anterior insulas* become thinner (Churchwell and Yurgelun-Todd, 2013), so they become less impulsive and plan more (Steinberg et al., 2008; Kasen et al., 2011). As a result, less impulsive, older students might be less susceptible to short-term Internet amusements and hence less likely to suffer from Internet addiction.

On the other hand, university students often move into a new environment without their family and old friends (Asendorpf, 2002). So, university students might feel lonelier and have less parent monitoring than otherwise, both of which might encourage impulsive university students to engage in more Internet activity and subsequently suffer from Internet addiction (Bozoglan et al., 2013; Yao et al., 2014). When students move away to university, they generally have fewer opportunities to keep their parents and childhood friends company, and making new friends might require substantial effort (Mattanah et al., 2010). As a result, they often miss their family (*homesickness*) and friends (*friendsickness*, Paul and Brier, 2001) and feel lonely (Larose and Boivin, 1998). Such lonely university students with impulsivity are easily tempted by the ready availability and immediate gratification of Internet entertainment. Without their parents or other family members nearby to monitor them (Henderson and Mapp, 2002), such impulsive university students might spend excessive time on the Internet and become addicted.

In summary, the research literature suggests two conflicting hypotheses. On the one hand, younger children are more impulsive than older children, more inclined to indulge in immediate Internet enjoyment excessively, and thus more likely to become addicted. On the other hand, older university students might feel lonely, use the Internet excessively without parent monitoring, and become addicted. As students with restraint are more disciplined and more focused on long-term goals, they are likely less susceptible to short-term amusements and Internet addiction. As few studies examined students at different ages, this meta-analysis can help determine whether age moderates the link between self-control and Internet addiction.

#### Gender

Unlike males, females pay more attention to their peers, monitor one another’s actions more, and are more influenced by one another (Rudman and Goodwin, 2004; Wills et al., 2004). In contrast, males are less attentive to their peers and less influenced by them, relying more on their judgments (Charness and Rustichini, 2011). As many males rely more on themselves than on their peers compared with females, males’ self-control is more likely to affect their behaviors and outcomes (Benda, 2013; Koon–Magnin et al., 2016), such as Internet addiction. Hence, we expect the link between self-control and Internet addiction to be stronger for males than for females.

#### Internet Addiction Measures and Year

In this study, we examine whether the following three Internet addiction measures moderate findings regarding the relationship between self-control and Internet addiction. Most past studies collected data on Internet addiction via surveys. For example, Young’s (1998b) Internet Addiction Test (IAT) comprises 20 items with a five-point scoring system, in which normal Internet users typically have a total score below 50 points. Higher total scores indicate greater severity of Internet addiction. Caplan’s (2002) Generalized Problematic Internet Use Scale (GPIUS) consists of 29 items with a five-point scoring system, capturing mood alteration, social benefits, negative outcomes, compulsivity, excessive time, withdrawal, and interpersonal control. Chen et al.’s (2003) Chinese Internet Addiction Scale (CIAS) has 26 items with a four-point scale and focuses on five constructs: compulsive use, withdrawal, tolerance, interpersonal relationship difficulties and health/time management difficulties. Other Internet addiction scales [e.g., Brenner’s (1997) Internet-Related Addictive Behavior Inventory (IRABI)] have not been widely used to study self-control and Internet addiction.

The internet has substantially changed over time, especially the increase in social media activities (Leung, 2014). Hence, we also test for differences across years.

### Purpose of This Study

This study aims to (a) synthesize the results of past studies on the relation between self-control and Internet addiction among students (10–22 years old) and (b) identify factors that influence this relationship. Specifically, this meta-analysis (a) calculates the overall effect size of the link between self-control and Internet addiction and (b) determines whether culture, age, gender, Internet addiction measures or publication year moderated this link.

## Materials and Methods

### Literature Search

This meta-analysis included Chinese- and English-language publications during January 2002 to September 2020. The Chinese-language articles were retrieved from the China Academic Journals Full-text Database, the China Selected Doctoral Dissertations and Masters’ Thesis Full-text Database, and Wanfang Data. We used the keywords ‘self-control (自我控制),’ ‘self-regulation (自我调节),’ and ‘impulse control (冲动控制).’ For Internet addiction, we used ‘Internet addiction (网络成瘾),’ ‘addiction (成瘾),’ ‘Internet abuse (网络滥用),’ and ‘Internet dependence (网络依赖).’

The English-language articles were retrieved from the Google Scholar, ProQuest Dissertations, Taylor Francis, Springer, Web of Science, PsycINFO, EBSCO, Elsevier SDOL. For self-control, we used the keywords ‘self-control,’ ‘self-regulation,’ ‘impulse control,’ and ‘impulse.’ For Internet addiction, we used ‘Internet addiction,’ ‘addiction,’ ‘Internet abuse,’ and ‘Internet dependence.’ These searches initially retrieved 279 articles.

### Study Selection via Inclusion and Exclusion Criteria

**FIGURE 1:**
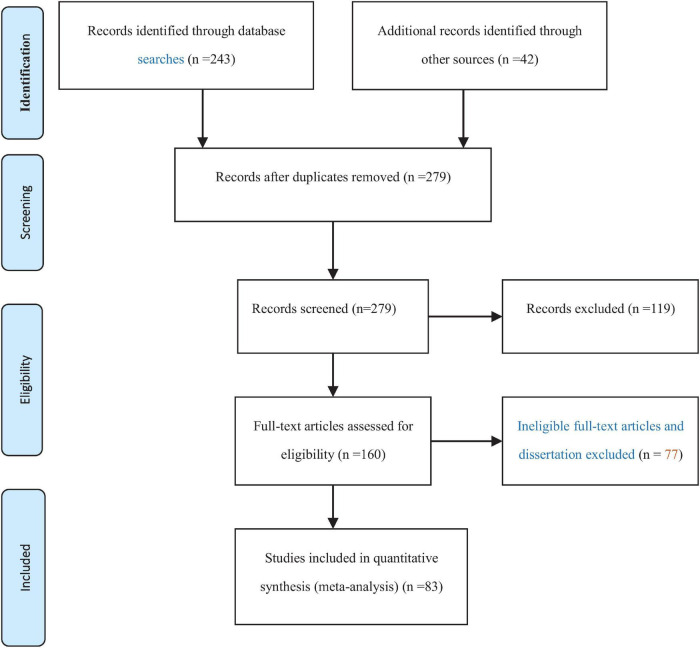
PRISMA 2009 flow diagram.

The articles were screened according to inclusion and exclusion criteria (Azer and Azer, 2015, see Figure 1). The inclusion criteria were: (a) it reported the relation between self-control and Internet addiction; (b) it reported either Pearson’s product-moment coefficients *r*, β, *T*, or *F*-values (the latter two can be converted to *r*-values); (c) it reported the sample size; (d) the sample predominantly comprised adolescents or university students; (e) when multiple publications use a data set, the result that used the complete data was used (based on reading the article titles, abstracts, and full text); (f) if multiple publications used the same data set, journal articles are preferred over conference proceedings, which were preferred over dissertations. The exclusion criteria were: (g) editorials, letters to the editor, opinion pieces, commentaries, personal views, and abstracts only, (h) review papers, (i) studies not written in English or Chinese.

We trained two reviewers to separately apply the inclusion and exclusion criteria to the studies. After the initial screening, 98 studies were identified by both reviewers. One reviewer also identified 6 additional studies. Both reviewers discussed the 6 studies, agreed to keep 4 of them, yielding a final set of 83 studies.

### Coding Variables

The collected articles were coded for the following features: internet addiction measures, author, publication year, sample size, proportion of female students, culture, ages, and *r* effect size (see Table 1). The studies used one of three Internet addiction measures: GPIUS (Caplan, 2002), IAT (Young, 1998b), CIAS (Chen et al., 2003), and others (e.g., IRABI). Furthermore, Internet addiction’s correlation with low self-control indicator or high self-control was encoded. If multiple effect sizes were obtained for the link between self-control and Internet addiction in the same sample, only the overall effect size was used. Also, the relation between self-control and Internet addiction was encoded among different groups of participants. If multiple methods were used to measure the relation between self-control and Internet addiction in the same study, the most statistically accurate one was used (e.g., β preferred over *r*). Comparison of the results using each encoding method showed high consistency.

**TABLE 1:** Characteristics of the 83 studies included in the meta-analysis.

Name	Culture^a^	*r*	Age^b^	*N* ^e^	Sorts	Female%	IA measures^c^	MERSQI score ^d^
Akın et al., 2015	2	−0.32	2	309	Restraint	51%	Others	9.0
Bai and Xu, 2009	1	0.51	2	427	Impulsivity	70%	Others	10.0
Błachnio and Przepiorka, 2016	2	0.04	3	284	Restraint	83%	CIAS	11.0
Blinka et al., 2015	2	0.24	1	18,709	Impulsivity	50%	IAT	9.0
Cai and Li, 2017	1	−0.56	2	682	Restraint	51%	GPIUS	9.5
Cao et al., 2018	1	−0.30	2	542	Restraint	45%	Others	11.0
Caplan, 2002	2	0.44	2	251	Impulsivity	70%	GPIUS	10.0
Caplan, 2002	2	−0.40	2	251	Restraint	70%	Others	10.0
Caplan, 2005	2	0.41	2	386	Impulsivity	70%	IAT	13.0
Chak and Leung, 2004	3	0.12	2	722	Impulsivity	64%	CIAS	9.0
Ching and Tak, 2016	1	−0.15	2	211	Restraint	*N*	Others	9.5
Choi et al., 2013	1	0.37	3	21	Impulsivity	55%	Others	11.0
Choi et al., 2014	1	0.78	2	23	Impulsivity	48%	IAT	10.0
Chou and Ting, 2003	2	0.76	3	1,395	Impulsivity	*N*	IAT	12.0
Davis et al., 2002	2	0.38	2	211	Impulsivity	51%	Others	11.0
Demetrovics et al., 2016	2	0.47	3	5,005	Impulsivity	49%	IAT	10.0
Du, 2013	1	−0.66	2	416	Restraint	54%	Others	11.0
Feng et al., 2014	1	0.48	2	192	Impulsivity	54%	GPIUS	12.0
Fırat, 2017	2	−0.27	2	60	Restraint	37%	IAT	9.0
Ge et al., 2010	1	−0.25	2	800	Restraint	*N*	Others	9.5
Geng et al., 2018	1	−0.47	2	405	Restraint	52%	Others	10.5
Gokçearslan et al., 2016	2	−0.25	2	614	Restraint	29%	CIAS	11.0
Gámez et al., 2015	2	0.23	1	801	Impulsivity	60%	GPIUS	10.0
He et al., 2012	1	−0.47	2	453	Restraint	0%	Others	9.0
Hou et al., 2013	1	0.21	2	942	Impulsivity	52%	Others	9.5
Iftikhar and Tariq, 2014	1	−0.10	2	100	Restraint	50%	Others	10.0
Iftikhar and Tariq, 2014	1	0.48	2	100	Impulsivity	50%	IAT	10.0
Ismail and Zawahreh, 2017	2	0.37	2	284	Impulsivity	50%	IAT	11.0
Jabutay and Kanthawongs, 2016	1	0.77	2	157	Impulsivity	43%	IAT	9.0
Jeong et al., 2016	3	−0.46	1	944	Restraint	49%	IAT	9.5
Jiang et al., 2018	1	0.26	1	2,056	Impulsivity	67%	Others	11.0
Jiang et al., 2017	1	−0.35	2	100	Restraint	53%	CIAS	9.0
Khang et al., 2013	2	−0.36	2	290	Restraint	64%	Others	9.5
Kim et al., 2017	1	−0.33	3	1,471	Restraint	17%	GPIUS	10.0
Kim et al., 2017	1	−0.14	2	377	Restraint	65%	Others	11.0
Kim et al., 2018	1	−0.30	1	665	Restraint	41%	CIAS	10.0
Larose and Eastin, 2010	2	0.27	2	218	Impulsivity	30%	Others	11.5
Larose et al., 2001	2	0.45	2	465	Impulsivity	39%	IAT	8.5
Lee and Cho, 2015	1	−0.03	1	93	Restraint	51%	CIAS	9.0
Lei et al., 2017	1	−0.53	2	543	Restraint	59%	Others	12.0
Li D. et al., 2013	1	−0.39	1	694	Restraint	55%	GPIUS	12.0
Li X. et al., 2013	1	−0.40	1	2,758	Restraint	54%	GPIUS	12.0
Li et al., 2014	1	0.30	1	966	Impulsivity	*N*	IAT	11.0
Li X. et al., 2016	1	0.45	2	220	Impulsivity	67%	GPIUS	10.0
Li Y. et al., 2016	1	−0.49	1	913	Restraint	54%	IAT	10.0
Li et al., 2018	1	−0.32	2	461	Restraint	64%	IAT	10.0
Li and Wang, 2013	1	0.31	1	1,186	Impulsivity	53%	IAT	9.0
Liang et al., 2016	1	0.38	3	2,361	Impulsivity	53%	IAT	11.0
Lin and Tsai, 2002	1	0.15	1	751	Impulsivity	20%	IAT	12.0
Liu and Yang, 2012	1	−0.33	2	170	Restraint	48%	CIAS	11.0
Lu, 2016	1	0.64	2	453	Impulsivity	44%	GPIUS	12.0
Mehroof and Griffiths, 2010	2	−0.02	2	123	Restraint	41%	IAT	9.0
Mei and Chai, 2013	1	0.40	1	1,552	Impulsivity	58%	CIAS	11.0
Mei et al., 2015	1	0.27	1	1,551	Impulsivity	58%	IAT	12.0
Mesgarani et al., 2013	1	−0.07	3	129	Restraint	*N*	IAT	11.0
Mottram and Fleming, 2009	2	0.39	2	272	Impulsivity	68%	Others	12.0
Nie et al., 2013	1	0.20	2	784	Impulsivity	51%	GPIUS	10.0
Ningtyas, 2012	1	−0.75	2	850	Restraint	*N*	Others	9.5
Öğütçü et al., 2016	2	0.03	1	246	Impulsivity	59%	Others	8.5
Özdemir et al., 2014	2	0.32	2	648	Impulsivity	34%	IAT	8.5
Park et al., 2014	1	−0.17	1	654	Restraint	46%	IAT	9.0
Park et al., 2016	2	−0.14	1	300	Restraint	82%	CIAS	9.5
Rahmani and Lavasani, 2011	1	0.24	2	179	Impulsivity	61%	CIAS	11.0
Romano et al., 2013	2	0.39	2	60	Impulsivity	55%	Others	10.0
Son et al., 2013	1	−0.35	3	344	Restraint	0%	IAT	12.0
Sun et al., 2011	1	−0.41	1	231	Restraint	59%	IAT	11.0
Sun et al., 2015	1	−0.69	2	443	Restraint	57%	IAT	10.0
Takao et al., 2009	1	0.19	2	444	Impulsivity	28%	GPIUS	10.0
Tang et al., 2015	1	0.32	2	966	Impulsivity	57%	Others	11.0
Tao, 2016	1	0.14	1	966	Impulsivity	52%	CIAS	9.5
Tao and Li, 2009	1	0.28	1	966	Impulsivity	47%	IAT	10.0
Teng et al., 2014	1	0.35	2	250	Impulsivity	0%	Others	11.0
Van Deursen et al., 2015	2	−0.37	3	386	Restraint	67%	IAT	12.0
Wan et al., 2015	1	0.84	2	1,183	Impulsivity	41%	CIAS	11.0
Wang, 2009	1	−0.41	2	1,104	Restraint	64%	IAT	11.0
Wang et al., 2017	1	0.40	3	4,313	Impulsivity	54%	Others	10.0
Wang et al., 2012	1	0.41	2	1,986	Impulsivity	51%	CIAS	10.0
Wu et al., 2009	1	0.37	2	528	Impulsivity	44%	IAT	10.5
Yun et al., 2016	1	−0.04	1	1,852	Impulsivity	55%	Others	10.5
Zhang et al., 2013	1	0.38	2	1,117	Impulsivity	38%	Others	9.5
Zhang et al., 2017	1	−0.45	2	661	Restraint	65%	Others	9.0
Zhou, 2017	1	0.04	2	330	Impulsivity	54%	IAT	10.0
Zhou and Zhou, 2017	1	−0.41	2	1,313	Restraint	57%	IAT	11.0
Zhou and Wang, 2017	1	0.22	2	222	Impulsivity	58%	Others	11.0
Zhou et al., 2015	1	−0.45	2	1,000	Restraint	58%	Others	10.0

*^a^Culture: 1 = East Asia, 2 = Western Europe/North America, 3 = Others;*

*^b^Age: 1 = Adolescent, 2 = university student, 3 = mixed;*

*^c^IA measures: IAT = Internet Addiction Test, GPIUS = Generalized Problematic Internet Use Scale, CIAS = Chinese Internet Addiction Scale;*

*^d^MERSQI = Medical Education Research Study Quality Instrument score;*

*^e^N = not report.*

We used the country of the study as a rough proxy for culture. Specifically, culture was coded as ‘East Asia,’ ‘Western Europe/North America,’ or ‘Others’ (Lei et al., 2018). ‘East Asia’ referred to studies of students in Asian countries such as China (including Hong Kong and Taiwan), South Korea, Indonesia, Thailand, Japan, Vietnam, Pakistan, and so on. ‘Western Europe/North America’ referred to students in European and North American countries such as the European Union (EU), Dutch, United Kingdom, the United States of America, Canada, Australia, and so on. ‘Others’ referred to students in Saudi Arabia, Iran, and so on. We also test for differences across countries within a culture. Age was coded as adolescent (10–17 years; Olds et al., 2009) or university (undergraduate) student (18–22 years). We also recorded the proportions of female students and male students. As the studies span nearly two decades, we test for differences across years.

### Assessment of Study Quality

The literature quality was assessed with the Medical Education Research Study Quality Instrument (MERSQI) (Cook and Reed, 2015). Although developed for medical education, MERSQI is discipline neutral and hence suitable for assessing the quality of non-medical education research (Jensen and Konradsen, 2018). The ten items of MERSQI cover six domains: (a) study design, (b) sampling, (c) type of data, (d) validity evidence for evaluation instrument scores, (e) data analysis, and (f) outcome. As each domain has a maximum score of 3, the maximum total score for a study is 18. The mean consensus MERSQI score was 10.322, with a standard deviation of 1.08 and a median score of 10.0, indicating fair overall study quality. Total consensus MERSQI scores for each paper are shown in Table 1.

### Meta-Analysis

In this standard meta-analysis, effect sizes between affective self-control and Internet addiction were calculated for each sample (Lipsey and Wilson, 2001). Then, we tested whether the links between affective self-control and Internet addiction were moderated by culture, age, Internet addiction measure, gender, or year.

### Effect Size Calculation

Meta-analysis of Pearson’s product-moment coefficients *r* yielded the effect size (Borenstein et al., 2010). Fisher’s *z*-transformation was applied to *r*, weighted by the sample size with 95% confidence intervals: Z = 0.5* ln [(1 + *r*)/(1 − *r*)], where the variance of *Z* is *V*_Z_ = 1/*n* − 3 and the standard deviation of *Z* is SE_Z_ = (1/*n* − 3)^0.5^. The effect size is *r*_z_ = (e^2 × *z*^ − 1)/(e^2 × *z*^ + 1). The confidence interval is computed as follows:

*r*_*U*_ = $\bar{r}$ + *Z*(1 − *a*) × *SE*_*es*_
$\bar{r_{L}}$ = $\bar{r}$ − *Z*(1 − *a*) × *SE*_*es*_
SE_*es*_ = √1/*ΣWi*
*W*_*i*_ = *n*_*i*_ − 3

The homogeneity calculation formula is computed as follows:

*Q*_*w*_ = *ΣWi* (*r*_*Zi*_ − $\bar{r}$)^2^
*I*^2^ = [*Q* − (*K* − *1*)]*/Q* × *100%.*

Moreover, this study follows the normative guidelines for interpreting the magnitude of a correlation proposed by Gignac and Szodorai (2016) with the quantitative investigation that has avoided the subjectivity of Cohen’s (1988) effect size guidelines: 0.10, 0.20, and 0.30 for small, medium, and large correlations.

### Data Processing and Analysis

We used the comprehensive meta-analysis software CMA 2.0^1^. To test our hypotheses, we calculated sample sizes (*k*), weighted effect sizes (*r*), and 95% confidence intervals. We tested whether the mean effect sizes of the studies differed significantly (*homogeneity* test) via Cochrane’s *Q* and *I*^2^ (Huedo-Medina et al., 2006). If *I*^2^ exceeds 75, the effect sizes show significant heterogeneity; then, a random-effects model would be more suitable than a fixed-effects model for this meta-analysis (in a random-effects model, the selected studies are treated as random samples from a larger population to help generalize the findings). Averaged weighted correlation coefficients (within- and between-inverse-variance weights) of independent samples were used to compute mean effect sizes. If the homogeneity test showed significant variance in effect sizes between different samples’ characteristics, we tested for moderators. When the homogeneity test was significant (*Q*_*BET*_ > 0.05), *post hoc* analysis confirmed the different groups statistically. To examine moderator effects, we used meta-ANOVA for categorical variables and meta-regression analysis for continuous variables.

## Results

### Effect Size and the Homogeneity Test

After filtering the literature, we used 83 independent samples and calculated 85 effect sizes (46 between impulsivity indicator and Internet addiction, and 39 between restraint indicator and Internet addiction). In all, 80,681 students participated in the studies reviewed; sample sizes of individual studies ranged from 21 to 18,709.

The results indicated that impulsivity was positively related to Internet addiction (*r* = 0.371) and restraint was negatively related to Internet addiction (*r* = −0.362). These effect sizes were sufficiently large for moderator analysis (shown in Table 2).

**TABLE 2:** Random model of correlations between self-control and Internet addiction.

	*k*	Mean *r*	95% CI for *r*		Homogeneity test			Tau-squared			Test of null hypothesis (two-tailed)
				
			LL	UL	*Q*(*r*)	*p*	*I* ^2^	Tau^2^	*SE*	Tau	*z*-value
Restraint indicator	46	0.371	0.311	0.427	2609.769	0.0	98.276	0.050	0.022	0.224	11.385***
Impulsivity indicator	39	−0.362	−0.414	−0.307	771.822	0.0	95.077	0.035	0.011	0.187	−12.106***

***p < 0.001.

**FIGURE 2:**
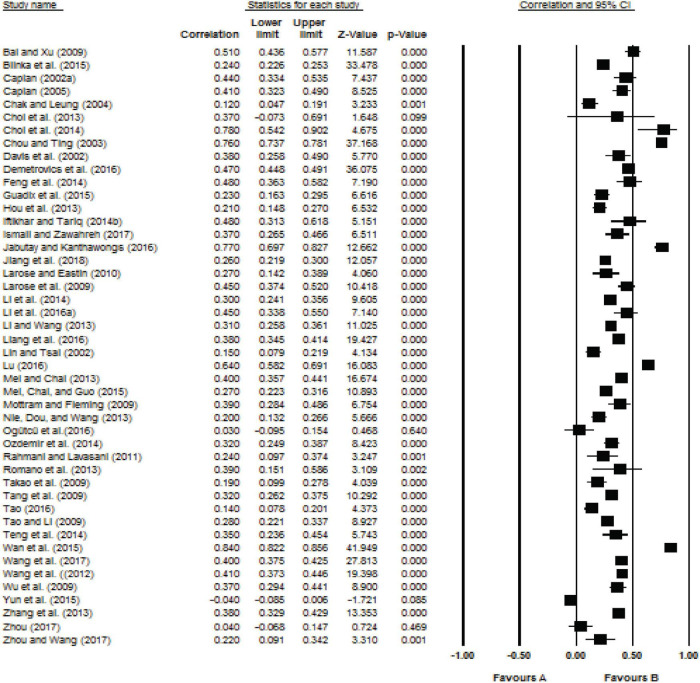
Forest plot for the relationship between impulsivity indicator and Internet addiction.

**FIGURE 3:**
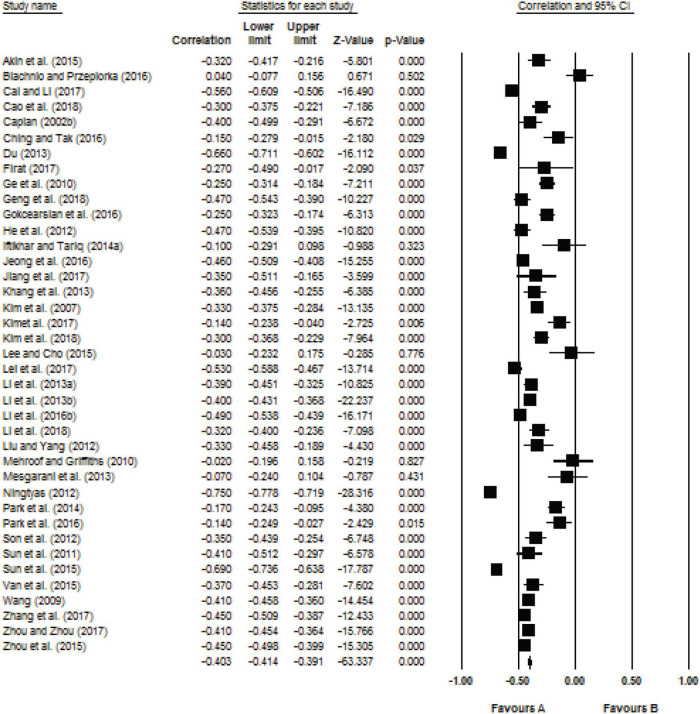
Forest plot for the relationship between restraint indicator and Internet addiction.

Forest plots summarize the results of all studies (using 95% confidence intervals of the standardized difference in means). See Figures 2, 3.

### Publication Bias

**FIGURE 4:**
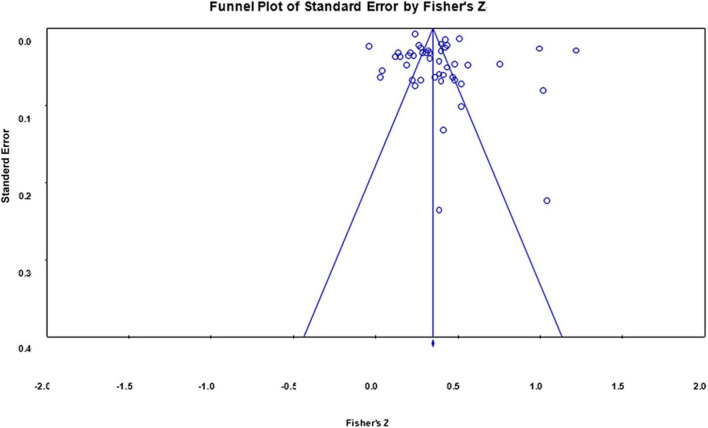
Funnel plot of effect sizes of the correlation between impulsivity indicator and Internet addiction.

**FIGURE 5:**
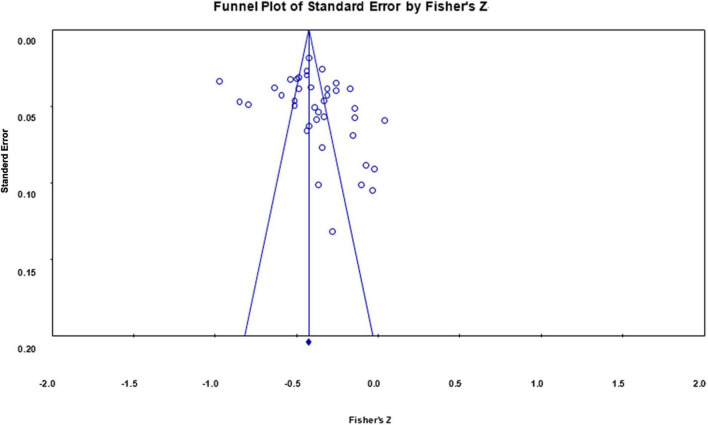
Funnel plot of effect sizes of the correlation between restraint indicator and Internet addiction.

We used funnel plots and Egger’s regressions (Egger et al., 1997) to test whether the results were biased. Both funnel plots of the correlation coefficients of impulsivity and restraint indicator with Internet addiction had symmetric distributions on both sides of the means, showing no publication bias (see Figures 4, 5).

Egger’s regressions for Internet addiction with both low self-control and high self-control indicators revealed no publication bias [*t*_(46)*LSCI*_ = 1.106, *p* = 0.273; *t*_(39)*HSCI*_ = 1.702, *p* = 0.097). Together, these findings suggested that the overall correlation between self-control and Internet addiction was stable, and provided no evidence of publication bias for these studies.

### Moderator Analysis

To test for moderators of the links between self-control and Internet addiction, we conducted two homogeneity tests, one across the 58 independent samples with impulsivity indicators and one across the 46 independent samples with restraint indicators. The results showed significant homogeneity coefficients and hence, significant moderation of the links between self-control indicators and Internet addiction [*Q*_*T*(46)Ipulsivity_ = 2609.769, *p* < 0.001; *Q*_*T*(39)Restraint_ = 771.822, *p* < 0.001). Next, we tested for moderators, using meta-ANOVA for categorical variables (internet addiction measure, culture [and separate countries], age) and meta-regression analysis for continuous variables (% female, year).

### Culture

The homogeneity coefficient showed that culture did not moderate the positive link between impulsivity indicator and Internet addiction (East Asia vs. Western Europe/North America, *Q*_*BET*_ = 1.294, *df* = 1, *p* > 0.05, see Table 3). However, the homogeneity test found significant differences in the correlation between restraint indicator and Internet addiction across the two cultures (*Q*_*BET*_ = 6.096, *df* = 1, *p* < 0.05); in this case, the link between a restraint indicator and Internet addiction was stronger in East Asia (*r* = −0.400) than in Western Europe/North America (*r* = −0.239). (Further analyses of studies by country showed no significant differences across countries within culture, results are available upon request).

**TABLE 3:** Culture and age moderated links between self-control and Internet addiction.

	Between-group effect (*Q*_*BET*_)	*k*	Mean *r*	*SE*	95% CI for *r*		Test of null (two-tailed) *z*-value within each group (Q_W_)
					LL	UL	

*Impulsivity indicator*							
**Culture**	1.254						
East Asian		31	0.374	0.020	0.295	0.447	8.731***
Western Europe/North America		14	0.383	0.051	0.266	0.489	6.061***
Others		1	0.120	0.000	−0.345	0.538	0.492
**Age**	12.572**						
Adolescent		12	0.218	0.008	0.106	0.325	3.758***
Undergraduate		29	0.408	0.030	0.342	0.470	11.001***
Mixed		5	0.510	0.037	0.358	0.636	5.843***
**Internet addiction *measures***	2.570						
CIAS		6	0.409	0.120	0.239	0.555	4.461***
GPIUS		7	0.386	0.032	0.225	0.527	4.466***
IAT		18	0.409	0.029	0.312	0.498	7.600***
Others		15	0.297	0.019	0.180	0.406	4.832***

***Restraint indicator***							

**Culture**	6.096*						
East Asian		27	−0.400	0.012	−0.457	−0.340	−11.836***
Western Europe/North America		10	−0.239	0.013	−0.355	−0.116	−3.753***
Others		2	−0.312	0.111	−0.532	−0.052	−2.333*
**Age**	4.548						
Adolescent		9	−0.324	0.011	−0.433	−0.207	−5.201***
Undergraduate		25	−0.400	0.016	−0.463	−0.334	−10.788***
Mixed		5	−0.228	0.023	−0.383	−0.060	−2.641**
**Internet addiction *measures***	7.626*						
CIAS		7	−0.198	0.013	−0.334	−0.055	−2.698***
GPIUS		4	−0.423	0.009	−0.561	−0.263	−4.840***
IAT		13	−0.363	0.014	−0.451	−0.268	−7.075***
Others		15	−0.410	0.026	−0.487	−0.326	−8.806***

*p < 0.05, **p < 0.01, ***p < 0.001.

### Age

The homogeneity test (*Q*_*BET*_ = 12.572, *df* = 2, *p* < 0.01) suggested that age moderated the link between impulsivity indicator and Internet addiction (see Table 3). The positive link between an impulsivity indicator and Internet addiction was significantly stronger for undergraduates (*r* = 0.408) than for adolescents (*r* = 0.218). However, the homogeneity test (*Q*_*BET*_ = 4.548, *df* = 1, *p* > 0.05) suggested that age did not moderate the link between restraint indicator and Internet addiction.

### Internet Addiction Measures

The homogeneity coefficient showed that Internet addiction measures did not moderate the relationship between impulsivity and Internet addiction (*Q*_*BET*_ = 2.570, *df* = 3, *p* > 0.05, see Table 3). However, the homogeneity test found significant differences in the link between high restraint indicator and Internet addiction across the Internet addiction measures (*Q*_*BET*_ = 7.626, *df* = 3, *p* > 0.05); in this case, the relationship was weaker among CIAS, compared to other internet addiction measures (| *r*_*CIAS*_| < | *r*_*IAT*_| < | *r*_*Others*_| < | *r*_*GPIUS*_| : | −0.198| < | −0.363| < | −0.410| < | −0.423|).

### Gender

To examine whether continuous variables (gender) moderated the links between self-control and Internet addiction, the *r* effect size was meta-regressed onto the percentage of female participants in each sample. Gender did not moderate the link between an impulsivity indicator and Internet addiction (*Q*_*Model*_ [1, *k* = 43] = 3.012, *p* > 0.05, see Table 4, top half). In contrast, gender moderated the link between a restraint indicator and Internet addiction (*Q*_*Model*_ [1, *k* = 33] = 12.220, *p* < 0.001), showing a stronger link for an all-male sample (*r* = −0.460) than an all-female sample (*r* = −0.300).

**TABLE 4:** Meta-regression analyses of gender and year.

	Variable	Parameter	Estimate	*SE*	*z*-value	95% CI for *b*	
						LL	UL
Impulsivity indicator	Female (%)	β_0_	0.391	0.021	16.74	0.340	0.431
		β_1_	−0.092	0.044	−2.101	−0.181	0.010
		*Q*_*Model*_ (1, *k* = 43) = 3.012, *p* > 0.05					
Restraint indicator	Female (%)	β_0_	−0.300	0.021	−12.673	−0.357	−0.265
		β_1_	−0.160	0.042	−3.552	−0.242	0.074
		*Q*_*Model*_ (1, *k* = 33) = 12.220, *p* < 0.01					
Impulsivity indicator	Year	β_0_	5.059	15.555	0.325	−25.427	35.545
		β_1_	−0.002	0.008	−0.300	0.017	0.013
		*Q*_*Model*_ (1, *k* = 45) = 0.090, *p* > 0.05					
Restraint indicator	Year	β_0_	−3.641	18.487	−0.197	−39.874	32.592
		β_1_	0.002	0.016	0.177	−0.016	0.020
		*Q*_*Model*_ (1, *k* = 38) = 0.031, *p* > 0.05					

### Year

Year was not a significant moderator. Year moderated neither the impulsivity indicator’s link with Internet addiction (*Q*_*Model*_ [1, *k* = 45] = 0.090, *p* > 0.05, see Table 4, bottom half) nor the restraint indicator’s link with Internet addiction (*Q*_*Model*_ [1, *k* = 38] = 0.031, *p* > 0.05).

## Discussion

As past studies of self-control and Internet addiction showed mixed results, this meta-analysis synthesized the results of such studies during 2002–2019. These results showed that restraint indicators were negatively linked to Internet addiction and that impulsivity indicators were positively linked to Internet addiction. Hence, self-control was negatively related to Internet addiction. Moreover, these links differed across culture, age, and gender. The negative link between a restraint indicator and Internet addiction was stronger for students in East Asia than in Western Europe/North America. Also, the positive link between an impulsivity indicator and Internet addiction was greater among university students than among adolescents. Lastly, the negative link between a restraint indicator and Internet addiction was stronger among males than females.

### Self-Control and Internet Addiction

Self-control was negatively linked to Internet addition, supporting Ainslie’s (1975) theory of impulsiveness. Specifically, these results are consistent with the view that students with more self-control than others are less impulsive (Peluso et al., 1999), and hence, less likely to engage in short-term behaviors that can yield Internet addiction and more likely to make short-term sacrifices/investment for long-term goals. Thus, these meta-analysis results suggest that self-control is an essential component of a comprehensive theory of Internet addiction (Lei et al., 2020). Moreover, this result suggests a possible intervention; specifically, future studies can determine whether interventions to enhance students’ self-control can reduce their Internet addiction.

### Moderation

Culture, age, gender, and Internet addiction measures moderated the links between self-control and Internet addiction. These results are consistent with short-term versus long-term orientation cultures, university student loneliness and reduced parent monitoring, and self-reliant males.

### Culture

Culture moderated Internet addiction’s link with restraint indicators but not its link with impulsivity indicators. The negative link between a restraint indicator and Internet addiction was stronger for students in East Asia than in Western Europe/North America. This result is consistent with the view that long-term orientation cultures (e.g., China; Hofstede et al., 2008) emphasize long-term goals, which largely supports students with high self-control to attend to and work toward them. By doing so, such students might further reduce their engagement with short-term Internet activities and hence are less likely to suffer from Internet addiction.

By contrast, the positive link between an impulsivity indicator and Internet addiction did not differ across cultures. This result is consistent with the view that impulsive students are less concerned with long-term goals (Metcalfe and Mischel, 1999). Hence, whether or not their country’s culture supports a long-term orientation does not affect their likelihood of Internet addiction.

These culture moderation results have theoretical and practical implications. First, any comprehensive theory of Internet addiction must include the moderation effect of culture. Also, if research shows that self-control interventions reduce Internet addictions, these culture moderation effects suggest that they might especially benefit not only impulsive students generally, but also self-disciplined students in short-term orientation cultures.

### Age

Age moderated the link between impulsivity indicators and Internet addiction. Compared with adolescents, university students showed a stronger positive link between impulsivity indicators and Internet addiction, but no moderation effect for restraint indicators. These results are consistent with the view that among university students who have transited into a new environment without their family and friends around, those with less restraint often have feelings of homesickness, friendsickness, or loneliness, and use the Internet excessively without parent monitoring, thereby becoming addicted (Larose and Boivin, 1998; Paul and Brier, 2001; Henderson and Mapp, 2002; Mattanah et al., 2010). Conversely, these results reject the claim that among students with low self-control, adolescents are more susceptible than university students to Internet addiction. Also, these results support the view that age does not moderate the negative link between restraint and Internet addiction.

In addition to being a vital component in a theory of Internet addiction, age is an important consideration for those exploring self-control interventions for reducing Internet addiction. Specifically, future studies can test whether interventions to improve self-control are especially effective for reducing Internet addiction among university students with impulsivity.

### Gender

Gender moderated the link between restraint indicator and Internet addiction. Specifically, the negative link between a restraint indicator and Internet addiction was stronger among males than females. This result partially supports the view that males are more self-reliant than females (Charness and Rustichini, 2011), the latter being more influenced by others (Rudman and Goodwin, 2004; Benda, 2013; Koon–Magnin et al., 2016); hence, greater self-control is linked to much less Internet addiction among males than among females. However, the positive link between an impulsivity indicator and Internet addiction did not differ by gender. Future studies that are more fine-grained can determine whether other mechanisms account for these different gender moderation results by restraint versus impulsivity indicators.

These significant gender results suggest that a comprehensive theory of Internet addiction must include gender differences. Furthermore, future studies can test whether interventions to improve self-control are especially effective for reducing Internet addiction not only among students with impulsivity but also among girls with restraint.

### Internet Addiction Measures

Internet addiction measures moderated the link between impulsivity and Internet addiction. The relationship was weaker among CIAS than other internet addiction measures. As China’s testing of Internet addiction was mostly restricted to CIAS, increasing the comparability of China’s results with other results worldwide entails more studies in China using GPIUS, IAT or other internet addiction measures rather than only CIAS.

### Limitations and Future Studies

The current study had several limitations, including a limited and biased sample, cross-sectional data, few moderating factors, and languages of studies. As the participants in this study were adolescents or university studies, future studies can include younger students and older adult students. Also, the number of studies in some subgroups show obvious difference, which may affect the robustness of subgroup analysis, so those results need to be interpreted cautiously and future research can include more relevant studies. Furthermore, we used Eastern versus Western countries as a rough proxy for culture. However, culture differs both across countries within a region (Germany vs. France; China vs. Japan) and within countries (Korean Americans vs. Irish Americans). Hence, future studies can test for culture effects more rigorously by asking participants to respond to culture questions. As the cross-sectional data in these studies cannot determine causation, future studies can collect more longitudinal data, including intervention studies that test possible solutions. As the current study only tested three types of moderating factors (internet addiction measures, culture, age, and gender), future studies can test other moderating factors, such as family attributes and peer relations. Moreover, only studies published in English and Chinese were used. As artificial intelligence improves translation software (e.g., Google translate), future meta-analyses can include published studies in more languages. Finally, this study that covers many factors, so it is not possible to focus in depth on any one of the aspects studied, we will depth explore the relationship between specific people’s self-control and Internet addiction in the future and focusing on one of the variables in order to analyze it in greater depth.

## Conclusion

Past studies of self-control and Internet addiction showed mixed results, so this meta-analysis synthesized 83 studies with 80,681 students to show that self-control was negatively linked to Internet addiction. Specifically, there was a positive link between impulsivity and Internet addiction, while there was a negative link between restraint and Internet addiction.

Furthermore, culture, age, gender, and Internet addiction measures moderated these links between self-control and Internet addiction. The negative link between a restraint indicator and Internet addiction was stronger in East Asia than in Western Europe/North America. Compared with adolescents, university students showed a greater positive link between impulsivity indicators and Internet addiction. Also, the negative link between a restraint indicator and Internet addiction was stronger among males than among females. Lastly, the relationship was weaker among CIAS than other Internet addiction measures.

## Data Availability Statement

The datasets presented in this study can be found in online repositories. The names of the repository/repositories and accession number(s) can be found in the article/supplementary material.

## Author Contributions

SL and HL provided the idea, designed the study, wrote the manuscript, and contributed to data collection. PR and MC provided the idea, designed the study, wrote the manuscript, and contributed to data analysis. CW contributed to design the study, analysed the data, and revised the manuscript. All authors approval of the version to be published and agreement to be accountable for all aspects of the work.

## Conflict of Interest

The authors declare that the research was conducted in the absence of any commercial or financial relationships that could be construed as a potential conflict of interest.

## Publisher’s Note

All claims expressed in this article are solely those of the authors and do not necessarily represent those of their affiliated organizations, or those of the publisher, the editors and the reviewers. Any product that may be evaluated in this article, or claim that may be made by its manufacturer, is not guaranteed or endorsed by the publisher.

## Footnotes

1
http://www.meta-analysis.com/

## References

[B1] Ainslie G. (1975). Specious reward: a behavioral theory of impulsiveness and impulse control. *Psychol. Bull.* 82 463–496. 10.1037/h0076860 1099599

[B2] Akın A. Arslan S. Arslan N. Uysal R. Sahranç Ü (2015). Self-control management and internet addiction. *Int. Online J. Educ. Sci.* 7 95–100. 10.15345/iojes.2015.03.016

[B3] Asendorpf J. B. (2002). “Shyness and adaptation to the social world of university,” in *Shyness*, ed. Crozier W. R. (Routledge), 119–136. 10.4324/9780203183427-13

[B4] Azer S. A. Azer D. (2015). Group interaction in problem-based learning tutorials: a systematic review. *Eur. J. Dental Educ.* 19 194–208. 10.1111/eje.12121 25327639

[B5] Bai Y. Xu Q. (2009). Research on the relationship between self-control ability and Internet overuse tendency of medical college students. *J. Qiqihar Med. College* 30 192–194.

[B6] Baumeister R. F. Gailliot M. Dewall C. N. Oaten M. (2010). Self-regulation and personality: how interventions increase regulatory success, and how depletion moderates the effects of traits on behavior. *J. Personality* 74 1773–1802. 10.1111/j.1467-6494.2006.00428.x 17083666

[B7] Benda B. (2013). Self-control, gender, and age: a survival analysis of recidivism among boot camp graduates in a 5-year follow-up. *J. Off. Rehabil.* 40 115–132. 10.1300/J076v40n03_06

[B8] Błachnio A. Przepiorka A. (2016). Dysfunction of self-regulation and self-control in facebook addiction. *Psychiatr. Quart.* 87 493–500. 10.1007/s11126-015-9403-1 26589423 PMC4945680

[B9] Blinka L. Skarupová K. Sevcíková A. Wolfling K. Müller K. W. Dreier M. (2015). Excessive internet use in European adolescents: what determines differences in severity? *Int. J. Public Health* 60 249–256. 10.1007/s00038-014-0635-x 25532555

[B10] Borenstein M. Hedges L. V. Higgins J. P. T. Rothstein H. R. (2010). A basic introduction to fixed-effect and random-effects models for meta-analysis. *Res. Synthesis Methods* 1 97–111. 10.1002/jrsm.12 26061376

[B11] Bozoglan B. Demirer V. Sahin I. (2013). Loneliness, self−esteem, and life satisfaction as predictors of internet addiction: a cross−sectional study among Turkish university students. *Scand. J. Psychol.* 54 313–319. 10.1111/sjop.12049 23577670

[B12] Brenner V. (1997). Psychology of computer use: XLVII. parameters of internet use, abuse and addiction: the first 90 days of the internet usage survey. *Psychol. Rep.* 80 879–882. 10.2466/pr0.1997.80.3.879 9198388

[B13] Cai C. Li J. (2017). The relationship between outcome expectation, network control self-efficacy and internet addiction in college students. *Technol. Wind* 3 168–169.

[B15] Cao Y. Qi J. Niu Z. Li C. Zhao A. Yi Y. (2018). Study on the effect of self-control on shopping addiction in college students. *Modern Med. Health Res.* 19:160.

[B16] Caplan S. E. (2002). Problematic internet use and psychosocial well-being: development of a theory-based cognitive–behavioral measurement instrument. *Comput. Hum. Behav.* 18 553–575. 10.1016/S0747-5632(02)00004-3

[B17] Caplan S. E. (2005). A social skill account of problematic internet use. *J. Commun.* 55 721–736. 10.1111/j.1460-2466.2005.tb03019.x

[B18] Carver C. S. (2005). Impulse and constraint: perspectives from personality psychology, convergence with theory in other areas, and potential for integration. *Personal. Soc. Psychol. Rev.* 9 312–333. 10.1207/s15327957pspr0904_2 16223354

[B19] Chak K. Leung L. (2004). Shyness and locus of control as predictors of internet addiction and internet use. *Cyberpsychol. Behav.* 7 559–570. 10.1089/cpb.2004.7.559 15667051

[B20] Charness G. Rustichini A. (2011). Gender differences in cooperation with group membership. *Games Econ. Behav.* 72 77–85. 10.1016/j.geb.2010.07.006

[B21] Chen S. H. Weng L. J. Su Y. J. Wu H. M. Yang P. F. (2003). Development of a Chinese internet addiction scale and its psychometric study. *Chin. J. Psychol.* 45 279–294.

[B22] Cheng C. Li A. Y. L. (2014). Internet addiction prevalence and quality of (real) life: a meta-analysis of 31 nations across seven world regions. *Cyberpsychol. Behav. Soc. Networking* 17 755–760. 10.1089/cyber.2014.0317 25489876 PMC4267764

[B23] Ching H. K. Tak L. M. (2016). The structural model in parenting style, attachment style, self-regulation and self-esteem with smartphone addiction. *IAFOR J. Psychol. Behav. Sci.* 3 85–103.

[B24] Choi J. S. Park S. M. Lee J. Hwang J. Y. Jung H. Y. Choi S. W. (2013). Resting-state beta and gamma activity in internet addiction. *Int. J. Psychophysiol.* 89 328–333. 10.1016/j.ijpsycho.2013.06.007 23770040

[B25] Choi J. S. Park S. M. Roh M. S. Lee J. Y. Park C. B. Hwang J. Y. (2014). Dysfunctional inhibitory control and impulsivity in internet addiction. *Psychiatry Res.* 215 424–428. 10.1016/j.psychres.2013.12.001 24370334

[B26] Chou C. Condron L. Belland J. C. (2005). A review of the research on internet addiction. *Educ. Psychol. Rev.* 17 363–388. 10.1007/s10648-005-8138-1

[B27] Chou T. J. Ting C. C. (2003). The role of flow experience in cyber-game addiction. *Cyberpsychol. Behav.* 6 663–675. 10.1089/109493103322725469 14756934

[B28] Churchwell J. C. Yurgelun-Todd D. A. (2013). Age-related changes in insula cortical thickness and impulsivity: significance for emotional development and decision-making. *Dev. Cogn. Neurosci.* 6 80–86. 10.1016/j.dcn.2013.07.001 23921157 PMC6987805

[B29] Cohen J. (1988). *Statistical Power Analysis for the Behavioral Sciences*, 2nd Edn. Boston: Erlbaum.

[B30] Cook D. A. Reed D. A. (2015). Appraising the quality of medical education research methods: the medical education research study quality instrument and the Newcastle–Ottawa scale-education. *Acad. Med.* 90 1067–1076. 10.1097/ACM.0000000000000786 26107881

[B31] Davis R. A. Flett G. L. Besser A. (2002). Validation of a new scale for measuring problematic internet use: implications for pre-employment screening. *Cyberpsychol. Behav.* 5 331–345. 10.1089/109493102760275581 12216698

[B32] DeLisi M. (2014). “Low self-control is a brain-based disorder,” in *The Nurture Versus Biosocial Debate in Criminology: On the Origins of Criminal Behavior and Criminality*, eds Beaver K. M. Barnes J. C. Boutwell B. B. (SAGE Publications), 172–184. 10.4135/9781483349114.n11

[B33] Demetrovics Z. Király O. Koronczai B. Griffiths M. D. Nagygyörgy K. Elekes Z. (2016). Psychometric properties of the problematic internet use questionnaire short-form (PIUQ-SF-6) in a nationally representative sample of adolescents. *PLoS One* 11:1–12. 10.1371/journal.pone.0159409 27504915 PMC4978438

[B34] Dewitte S. Cremer D. D. (2001). Self-control and cooperation: different concepts, similar decisions? A question of the right perspective. *J. Psychol.* 135 133–153. 10.1080/00223980109603686 11403337

[B35] Du L. (2013). The relationship between network self-control ability and pathological internet use in college students. *Chin. J. School Health* 8 990–991.

[B36] Duckworth A. L. Steinberg L. (2015). Unpacking self−control. *Child Dev. Perspect.* 9 32–37. 10.1111/cdep.12107 25821515 PMC4372146

[B37] Dvorak R. D. Simons J. S. (2009). Moderation of resource depletion in the self-control strength model: differing effects of two modes of self-control. *Personal. Soc. Psychol. Bull.* 35 572–583. 10.1177/0146167208330855 19193603

[B38] Egger M. Smith G. D. Schneider M. Minder C. (1997). Bias in meta-analysis detected by a simple, graphical test. *Br. Med. J.* 315 629–634. 10.1136/bmj.315.7109.629 9310563 PMC2127453

[B39] Feng J. Zeng S. Sun Y. Kenichi K. (2014). A study on the relationship between undergraduates’ internet addiction disorder, online game experience, online time and sleep quality. *World J. Sleep Med.* 4 193–197.

[B40] Fırat M. (2017). Relationship between self-control and facebook use: case of CEIT students. *Educ. Sci. Theory Practice* 17 1179–1201.

[B41] Gámez M. Calvete E. Orue I. Las Hayas C. (2015). Problematic internet use and problematic alcohol use from the cognitive–behavioral model: a longitudinal study among adolescents. *Addictive Behav.* 40 109–114. 10.1016/j.addbeh.2014.09.009 25244690

[B42] Gao Y. Zhao L. (2009). On upgrading college students ‘mental resilience and measures of handling network addiction. *J. Jilin Province Econ. Manage. Cadre College* 23 115–117.

[B43] Ge L. Ge X. Zhao J. (2010). Correlation between locus of control and internet addiction in college students. *China J. Health Psychol.* 11 1386–1387.

[B44] Geng J. Han L. Gao F. Zou M. Huang C. (2018). Internet addiction and procrastination among Chinese young adults: a moderated mediation model. *Computers in Human Behavior* 84 320–333. 10.1016/j.chb.2018.03.013

[B45] Gignac G. E. Szodorai E. T. (2016). Effect size guidelines for individual differences researchers. *Personal. Indiv. Diff.* 102 74–78. 10.1016/j.paid.2016.06.069

[B46] Gokçearslan S. Mumcu F. Haçlaman T. Çevik Y. D. (2016). Modelling smartphone addiction: the role of smartphone usage, self-regulation, general self-efficacy and cyberloafing in university students. *Comput. Hum. Behav.* 63 639–649. 10.1016/j.chb.2016.05.091

[B48] He C. Xia M. Jiang G. Wei H. (2012). Mediation role of self-control between internet game addiction and self-esteem. *Chin. J. Clin. Psychol.* 20 58–60.

[B49] Henderson A. T. Mapp K. L. (2002). A new wave of evidence: the impact of school, family and community connections on student achievement. *Natl. Center Family Commun. Connect. Schools* 97, 13–14.

[B50] Hofmann W. Friese M. Strack F. (2009). Impulse and self-control from a dual-systems perspective. *Perspect. Psychol. Sci.* 4 162–176. 10.1111/j.1745-6924.2009.01116.x 26158943

[B51] Hofstede G. J. Jonker C. M. Verwaart T. (2008). “Long-term orientation in trade,” in *Complexity and Artificial Markets*, eds Hauser F. Schredelseker K. (Springer), 107–119. 10.1007/978-3-540-70556-7_9

[B52] Hou Z. Zhang Q. Yang G. (2013). Effects of different addiction states on activities, personalities and self-control ability among undergraduate internet user. *J. Zhejiang Univ.* 40 106–111.

[B53] Huedo-Medina T. B. Sánchez-Meca J. Marin-Martinez F. Botella J. (2006). Assessing heterogeneity in meta-analysis: Q statistic or I^2^ index? *Psychol. Methods* 11 193–206. 16784338 10.1037/1082-989X.11.2.193

[B54] Hur M. H. (2006). Demographic, habitual, and socioeconomic determinants of Internet addiction disorder: an empirical study of Korean teenagers. *Cyberpsychol. Behav.* 9 514–525. 10.1089/cpb.2006.9.514 17034317

[B55] Iftikhar M. Tariq S. (2014). Self-control, narcissistic tendencies and internet addiction among adolescents. *J. Arts Soc. Sci.* 4 1–17.

[B56] Ismail A. B. Zawahreh N. (2017). Self-control and its relationship with the internet addiction among a sample of najran university students. *J. Educ. Hum. Dev.* 6 168–174.

[B57] Jabutay F. Kanthawongs P. (2016). “An empirical study on the impact of self–regulation and compulsivity towards smartphone addiction of university students,” in *Proceedings of 2016 International Conference on Cognition and Exploratory Learning in the Digital Age*, (International Association for Development of the Information Society), 28–30.

[B58] Jensen L. Konradsen F. (2018). A review of the use of virtual reality head-mounted displays in education and training. *Educ. Inf. Technol.* 23, 1515–1529. 10.1007/s10639-017-9676-0

[B59] Jeong S. H. Kim H. J. Yum J. Y. Hwang Y. (2016). What type of content are smartphone users addicted to? SNS vs. games. *Comput. Hum. Behav.* 54 10–17. 10.1016/j.chb.2015.07.035

[B60] Jiang Y. Wang H. Jiang H. Liu Y. (2018). The influence of neuroticism on the excessive use of mobile social networks in adolescents: the dual mediating effects of impulsive and interpersonal disturbance. *Stud. Psychol. Behav.* 16 130–140.

[B61] Jiang Z. Zhao X. Li C. (2017). Self–control predicts attentional bias assessed by online shopping–related stroop in high online shopping addiction tendency college students. *Comprehensive Psychiatry* 75 14–21. 10.1016/j.comppsych.2017.02.007 28284828

[B62] Kasen S. Cohen P. Chen H. (2011). Developmental course of impulsivity and capability from age 10 to age 25 as related to trajectory of suicide attempt in a community cohort. *Suicide Life Threat. Behav.* 41 180–192. 10.1111/j.1943-278X.2011.00017.x 21342218 PMC3082462

[B63] Khang H. Kim J. K. Kim Y. (2013). Self–traits and motivations as antecedents of digital media flow and addiction: the internet, mobile phones, and video games. *Comput. Hum. Behav.* 29 2416–2424. 10.1016/j.chb.2013.05.027

[B64] Kim E. J. Namkoong K. Ku T. Kim S. J. (2008). The relationship between online game addiction and aggression, self–control and narcissistic personality traits. *European Psychiatry* 23 212–218. 10.1016/j.eurpsy.2007.10.010 18166402

[B65] Kim J. Hong H. Lee J. Hyun M. H. (2017). Effects of time perspective and self–control on procrastination and internet addiction. *J. Behav. Addict.* 6 229–236. 10.1556/2006.6.2017.017 28494615 PMC5520116

[B66] Kim Y. J. Roh D. Lee S. K. Canan F. Potenza M. N. (2018). Factors statistically predicting at–risk/problematic Internet use in a sample of young adolescent boys and girls in South Korea. *Front. Psychiatry* 9:1–9. 10.3389/fpsyt.2018.00351 30131728 PMC6090057

[B67] Koon–Magnin S. Bowers D. Langhinrichsen–Rohling J. Arata C. (2016). Social learning, self–control, gender, and variety of violent delinquency. *Deviant Behavior* 37 824–836. 10.1080/01639625.2016.1147798

[B68] Lam L. T. Peng Z. W. (2010). Effect of pathological use of the internet on adolescent mental health: a prospective study. *Arch. Pediatr. Adolescent Med.* 164 901–906. 10.1001/archpediatrics.2010.159 20679157

[B69] Larose R. Eastin M. S. (2010). Is online buying out of control? Electronic commerce and consumer self–regulation. *J. Broad. Electr. Media* 46 549–564. 10.1207/s15506878jobem4604_4

[B70] Larose R. Mastro D. Eastin M. S. (2001). Understanding internet usage, a social-cognitive approach to uses and gratifications. *Soc. Sci. Comput. Rev.* 19, 395–413. 10.1177/089443930101900401

[B71] Larose R. Lin C. A. Eastin M. S. (2003). Unregulated internet usage: addiction, habit, or deficient self–regulation? *Media Psychol.* 5 225–253.

[B72] Larose S. Boivin M. (1998). Attachment to parents, social support expectations, and socioemotional adjustment during the high school–college transition. *J. Res. Adolescence* 8 1–27. 10.1207/s15327795jra0801_1

[B73] Lee J. Cho B. (2015). Effects of self–control and school adjustment on smartphone addiction among elementary school students. *Int. J. Contents* 11 1–6. 10.5392/IJoC.2015.11.3.001

[B74] Lei H. Jing G. Min J. Feng G. Hua Y. (2017). Relationship between shyness and mobile phone addiction in Chinese young adults: mediating roles of self-control and attachment anxiety. *Comput. Hum. Behav.* 76, 363–371. 10.1016/j.chb.2017.07.036

[B75] Lei H. Chiu M. M. Cui Y. Zhou W. Li S. (2018). Parenting style and aggression: a meta–analysis of mainland Chinese children and youth. *Child. Youth Serv. Rev.* 94 446–455. 10.1016/j.childyouth.2018.07.033

[B76] Lei H. Mao W. Cheong C. M. Wen Y. Cui Y. Cai Z. (2020). The relationship between self-esteem and cyberbullying: a meta-analysis of children and youth students. *Curr. Psychol.* 3 830–842. 10.1007/s12144-019-00407-6

[B77] Leung L. (2014). Predicting internet risks: a longitudinal panel study of gratifications–sought, internet addiction symptoms, and social media use among children and adolescents. *Health Psychol. Behav. Med. Open Access J.* 2 424–439. 10.1080/21642850.2014.902316 25750792 PMC4346000

[B78] Li B. Shi Z. Wang S. Zheng K. Zhao X. (2018). The correlation between college students’ degree of wechat use and their loneliness: the mediating role of self–control. *Psychol. Techn. Applic.* 6 51–57.

[B79] Li C. Dang J. Zhang X. Zhang Q. Guo J. (2014). Internet addiction among Chinese adolescents: the effect of parental behavior and self–control. *Comput. Hum. Behav.* 41 1–7. 10.1016/j.chb.2014.09.001

[B80] Li D. Li X. Wang Y. Zhao L. Bao Z. Wen F. (2013). School connectedness and problematic internet use in adolescents: a moderated mediation model of deviant peer affiliation and self–control. *J. Abnormal Child Psychol.* 41 1231–1242. 10.1007/s10802-013-9761-9 23695186

[B81] Li J. Wang W. (2013). The characteristics of internet addiction among high school freshmen in Chongqing and its relationship with impulsiveness. *China J. Health Psychol.* 21 420–422.

[B82] Li X. Li D. Newman J. (2013). Parental behavioral and psychological control and problematic internet use among Chinese adolescents: the mediating role of self–control. *Cyberpsychol. Behav. Soc. Networking* 16 442–447. 10.1089/cyber.2012.0293 23509987

[B83] Li X. Xin T. Zhang Y. Du Y. Liu Y. Jiang Y. (2016). Boredom proneness and mobile phone addiction: mediating of self–control. *Chin. J. School Health* 37 1487–1490.

[B84] Li Y. Jin L. Zhang S. (2016). Research on the relationship between self–control ability and mobile phone dependence among university students. *Chin. J. Health Educ.* 32 775–778+802.

[B85] Liang H. Wang L. Fan C. (2016). Relationship between the due self–control system and internet addiction among adolescents. *Chin. General Practice* 19 1076–1080.

[B86] Lin S. Tsai C. C. (2002). Sensation seeking and internet dependence of Taiwan high school adolescents. *Comput. Hum. Behav.* 18 411–426. 10.1016/S0747-5632(01)00056-5

[B87] Lipsey M. W. Wilson D. B. (2001). *Practical Meta-Analysis.* Thousand Oaks, CA: SAGE Publications.

[B88] Liu H. Yang N. (2012). Correlation study on internet addiction, time management disposition and anxiety form among undergraduates. *Soc. Psychol. Sci.* 141 92–95.

[B89] Lu X. (2016). The current situation of network dependence and the influence of self–control on students—based on the empirical study of Sino–foreign cooperative education in Jiangsu province. *J. Jiangsu Second Normal Univ.* 9 30–36.

[B90] Maloney P. W. Grawitch M. J. Barber L. K. (2012). The multi-factor structure of the brief self-control scale: discriminant validity of restraint and impulsivity. *J. Res. Personal.* 46 111–115. 10.1016/j.jrp.2011.10.001

[B91] Mattanah J. F. Ayers J. F. Brand B. L. Brooks L. J. Quimby J. L. McNary S. W. (2010). A social support intervention to ease the college transition: exploring main effects and moderators. *J. College Stud. Dev.* 51 93–108. 10.1353/csd.0.0116 34409987

[B92] Mehroof M. Griffiths M. D. (2010). Online gaming addiction: the role of sensation seeking, self–control, neuroticism, aggression, state anxiety, and trait anxiety. *Cyberpsychol. Behav. Soc. Networking* 13 313–316. 10.1089/cyber.2009.0229 20557251

[B93] Mei S. Chai J. (2013). Teenagers’ access to the Internet with cell phones and its relationship with their subjective well–being and self–control. *Chin. J. Special Educ.* 159 80–85.

[B94] Mei S. Chai J. Guo J. (2015). Subjective well–being and internet addiction of adolescents: mediating roles of self–esteem and self–control. *Psychol. Dev. Educ.* 31 603–609.

[B95] Mesgarani M. Shafiee S. Ahmadi E. Zare F. (2013). Studying the relationship between internet addiction and emotional intelligence, sensation seeking and metacognition among those who referred to cafes. *Int. Res. J. Appl. Basic Sci.* 4 889–893.

[B96] Metcalfe J. Mischel W. (1999). A hot/cool-system analysis of delay of gratification: dynamics of willpower. *Psychol. Rev.* 106 3–19. 10.1037/0033-295X.106.1.3 10197361

[B97] Mottram A. J. Fleming M. J. (2009). Extraversion, impulsivity, and online group membership as predictors of problematic internet use. *Cyberpsychol. Behav.* 12 319–321. 10.1089/cpb.2007.0170 19445635

[B98] Nie Y. Dou K. Wang Y. (2013). Impulsive and internet addition disorder: a mediator role of self–control. *J. Ningbo Univ.* 3 7–12.

[B99] Ningtyas D. (2012). Hubungan antara self control dengan internet addiction pada mahasiswa program studi piskologi universitas sebelas maret surakarta. *J. Ilmiah Psikologi Candrajiwa* 1 25–30.

[B100] Öğütçü G. Çırakoğlu O. C. Cula S. (2016). Information security in the world of digital natives: how internet addiction, sensation seeking and information security behaviors are related. *Int. J. Manage. Appl. Sci.* 83 79–84. 10.1016/j.cose.2015.10.002

[B101] Olds T. Wake M. Patton G. Ridley K. Waters E. Williams J. (2009). How do school–day activity patterns differ with age and gender across adolescence? *J. Adolescent Health* 44 64–72. 10.1016/j.jadohealth.2008.05.003 19101460

[B102] Özdemir Y. Kuzucu Y. Ak S. (2014). Depression, loneliness and internet addiction: how important is low self–control? *Comput. Hum. Behav.* 34 284–290. 10.1016/j.chb.2014.02.009

[B103] Park J. A. Park M. H. Shin J. H. Li B. Rolfe D. T. Yoo J. Y. (2016). Effect of sports participation on Internet addiction mediated by self–control: a case of korean adolescents. *Kasetsart J. Soc. Sci.* 37 164–169. 10.1016/j.kjss.2016.08.003

[B104] Park S. Kang M. Kim E. (2014). Social relationship on problematic internet use (PIU) among adolescents in South Korea: a moderated mediation model of self–esteem and self–control. *Comput. Hum. Behav.* 38 349–357. 10.1016/j.chb.2014.06.005

[B105] Paul E. L. Brier S. (2001). Friendsickness in the transition to college: precollege predictors and college adjustment correlates. *J. Counseling Dev.* 79 77–89. 10.1002/j.1556-6676.2001.tb01946.x

[B106] Peluso T. Ricciardelli L. A. Williams R. J. (1999). Self–control in relation to problem drinking and symptoms of disordered eating. *Addict. Behav.* 24 439–442. 10.1016/S0306-4603(98)00056-2 10400283

[B107] Ponton M. K. Rhea N. E. (2006). Autonomous learning from a social cognitive perspective. *New Horizons Adult Educ. Hum. Resour. Dev.* 20 38–49.

[B108] Rahmani S. Lavasani M. G. (2011). The relationship between internet dependency with sensation seeking and personality. *Proc. Soc. Behav. Sci.* 30 272–277. 10.1016/j.sbspro.2011.10.054

[B109] Romano M. Osborne L. A. Truzoli R. Reed P. (2013). Differential psychological impact of internet exposure on internet addicts. *PLos One* 8:1–6. 10.1371/journal.pone.0055162 23408958 PMC3567114

[B110] Rosen L. D. Cheever N. A. Carrier L. M. (2014). “Social networking is addictive and can lead to psychological disorders,” in *Are Social Networking Sites Harmful?*, ed. Berlatsky N. (Greenhaven Press), 51–58.

[B111] Rudman L. A. Goodwin S. A. (2004). Gender differences in automatic in–group bias: why do women like women more than men like men? *J. Personal. Soc. Psychol.* 87:494. 10.1037/0022-3514.87.4.494 15491274

[B112] Shaw M. Black D. W. (2008). Internet addiction. *CNS Drugs* 22 353–365. 10.2165/00023210-200822050-00001 18399706

[B113] Shek D. T. L. Lu Y. (2012). Internet addiction phenomenon in early adolescents in Hong Kong. *Sci. World J.* 2012:104304. 10.1100/2012/104304 22778694 PMC3385635

[B114] Son D. T. Yasuoka J. Poudel K. C. Otsuka K. Jimba M. (2013). Massively multiplayer online role–playing games (MMORPG): association between its addiction, self–control and mental disorders among young people in Vietnam. *Int. J. Soc. Psychiatry* 59 570–577. 10.1177/0020764012445861 22718852

[B115] Steinberg L. Albert D. Cauffman E. Banich M. Graham S. Woolard J. (2008). Age differences in sensation seeking and impulsivity as indexed by behavior and self–report: evidence for a dual systems model. *Dev. Psychol.* 44:1764. 10.1037/a0012955 18999337

[B116] Strack F. Deutsch R. (2004). Reflective and impulsive determinants of social behavior. *Personal. Soc. Psychol. Rev.* 8 220–247. 10.1207/s15327957pspr0803_1 15454347

[B117] Sun G. Yu Y. Luo Z. Li Y. Zhao X. (2011). Research on middle school students’ mobile phone internet addition and self–control over internet use. *China J. Health Psychol.* 9 1078–1080.

[B118] Sun X. Zhao X. Zhou Z. Chen W. Bao N. (2015). Mediation role of self–control in internet use between time management disposition and pathological internet use. *Stud. Psychol. Behav.* 13 410–413.

[B119] Takao M. Takahashi S. Kitamura M. (2009). Addictive personality and problematic mobile phone use. *Cyberpsychol. Behav.* 12 501–507. 10.1089/cpb.2009.0022 19817562

[B120] Tang Y. Zou J. Li M. Liang J. Liu W. (2015). Subjective well–being and mobile phone dependence among vocational college students: the mediating role of self–esteem and self–control. *Chin. J. School Doctor* 721–725.

[B121] Tao Y. (2016). Analysis of the influence of self–control on adolescent internet addiction. *New West* 125–126.

[B122] Tao Y. Li C. (2009). Research on the mediating effect of self–control on internet addiction disorder and parental rearing style. *China J. Health Psychol.* 17 1444–1447.

[B123] Teng Z. Griffiths M. D. Nie Q. Xiang G. Guo C. (2020). Parent–adolescent attachment and peer attachment associated with internet gaming disorder: a longitudinal study of first-year undergraduate students. *J. Behav. Addic.* 9 116–128. 10.1556/2006.2020.00011 32359235 PMC8935186

[B124] Teng Z. Li Y. Liu Y. (2014). Online gaming, internet addiction, and aggression in chinese male students: the mediating role of low self–control. *Int. J. Psychol. Stud.* 6 88–97. 10.5539/ijps.v6n2p89

[B125] Throuvala M. A. Janikian M. Griffiths M. D. Rennoldson M. Kuss D. J. (2019). The role of family and personality traits in internet gaming disorder: a mediation model combining cognitive and attachment perspectives. *J. Behav. Addic.* 8 48–62. 10.1556/2006.8.2019.05 30739463 PMC7044602

[B126] Tsitsika A. Janikian M. Schoenmakers T. M. Tzavela E. C. Olafsson K. Wójcik S. (2014). Internet addictive behavior in adolescence: a cross–sectional study in seven European countries. *Cyberpsychol. Behav. Soc. Networking* 17 528–535. 10.1089/cyber.2013.0382 24853789

[B127] Van Deursen A. J. A. M. Bolle C. L. Hegner S. M. Kommers P. A. M. (2015). Modeling habitual and addictive smartphone behavior. *Comput. Hum. Behav.* 45 411–420. 10.1016/j.chb.2014.12.039

[B128] Wan J. Liu L. Fang X. (2015). On the relationship between college students’ psychological needs or self–efficacy and internet addiction. *Chin. J. Special Educ.* 3 86–91.

[B129] Wang J. You Y. Huang J. Chen D. Zhuang H. (2012). The relationship between internet addiction and ego–identity, self–control ability of college students. *J. Psychiatry* 25 350–352.

[B130] Wang L. Tao T. Fan C. Gao W. Wei C. (2017). The association between internet addiction and both impulsivity and effortful control and its variation with age. *Addic. Res. Theory* 25 83–90. 10.1080/16066359.2016.1206082

[B131] Wang Y. (2009). Relationship between college students’ self –regulation and the tendency of internet addiction. *Educ. Teach. Res.* 23 37–39.

[B132] Wills T. A. Resko J. A. Ainette M. G. Mendoza D. (2004). Role of parent support and peer support in adolescent substance use: a test of mediated effects. *Psychol. Addic. Behav.* 18:122. 10.1037/0893-164X.18.2.122 15238054

[B133] Wu Y. An Y. Hou S. (2009). A study on the relationship between IAD and locus of control among university students. *J. Shanxi Agric. Univ.* 8 329–332.

[B134] Yao M. Z. He J. Ko D. M. Pang K. (2014). The influence of personality, parental behaviors, and self-esteem on Internet addiction: a study of Chinese college students. *Cyberpsychol. Behav. Soc. Networking* 17 104–110. 10.1089/cyber.2012.0710 24003966 PMC3924803

[B135] Young K. S. (1998a). Caught in the net: how to recognize the signs of internet addictionand a winning strategy for recovery. *Assessment* 21 713–722.

[B136] Young K. S. (1998b). Internet addiction: the emergence of a new clinical disorder. *CyberPsychol. Behav.* 1 237–244.

[B137] Young K. S. (2004). Internet addiction: a new clinical phenomenon and its consequences. *Am. Behav. Sci.* 48 402–415. 10.1177/0002764204270278

[B138] Yun I. Kim S. G. Kwon S. (2016). Low self–control among south Korean adolescents: a test of Gottfredson and Hirschi’s generality hypothesis. *Int. J. Off. Ther. Comparat. Criminol.* 60 1185–1208. 10.1177/0306624X15574683 25814317

[B139] Zhang X. Zhang Y. Wu M. (2017). Research on the relationship between self–control and cell phone dependence in college students. *J. Lanzhou Instit. Educ.* 9 158–160.

[B140] Zhang Y. Zhang W. Wu L. (2013). Relationship between mobile phone internet addiction and impulsivity among medical college students. *China J. Health Psychol.* 21 284–287.

[B141] Zhou E. Zhou H. (2017). An empirical study of college student’s subjective well–being, self–control and internet addiction. *J. Gradual School Chin. Acad. Soc. Sci.* 19–26.

[B142] Zhou H. (2017). Study on vocational college students cell phone addiction and self–control. *J. Lanzhou Petrochem. Polytechnic* 3 52–56.

[B143] Zhou X. Wang X. (2017). Relationship between we chat addiction and self–control in college students. *China J. Health Psychol.* 8 1254–1257.

[B144] Zhou Y. Liu Y. Chen J. (2015). Mediation role of self–control between mobile phone addiction and self–esteem. *Chin. J. School Health* 36 1032–1034.

